# Historical ecology meets conservation and evolutionary genetics: a secondary contact zone between *Carabus violaceus* (Coleoptera, Carabidae) populations inhabiting ancient and recent woodlands in north-western Germany

**DOI:** 10.3897/zookeys.100.1546

**Published:** 2011-05-20

**Authors:** Andrea Matern, Claudia Drees, Werner Härdtle, Goddert von Oheimb, Thorsten Assmann

**Affiliations:** 1 Institute of Ecology and Environmental Chemistry, Leuphana University Lüneburg, Scharnhorststraße 1, D – 21335 Lüneburg, Germany; 2 Tel Aviv University, George S. Wise Faculty of Life Sciences, Department of Zoology, The National Collections of Natural History, Tel Aviv 69978, Israel

**Keywords:** fragmentation, afforestation, allozymes, morphometrics, *Carabus violaceus purpurascens*, hybridization, hybrid zone

## Abstract

Only very few cases have documented that an increase in connectivity after a period of fragmentation in ecological time has had an effect on the distribution, genetic structure and morphology of stenotopic species. In this study we present an example of clinal variability in a woodland ground beetle as a result of changes in the connectivity of a landscape during the last two centuries. The study area hosts both the nominate form *Carabus violaceus* s. str. and the subspecies *Carabus violaceus purpurascens*, which is ranked as a distinct species by some authors. We studied 12 *Carabus violaceus* populations from a 30 km transect of ancient and recent forests in north-western Germany. We analyzed three polymorphic enzyme loci, classified the elytron sculpture and measured the shape of the aedeagus tip of the specimens. *Carabus violaceus* showed secondary gradients both in allozyme markers and morphometric characters in our study area. A genetic differentiation of 16% between the populations is high but lies within the range of intraspecific variability in habitat specialists of the genus *Carabus*. Populations had no significant deficit of heterozygotes. We found many hybrid populations in terms of morphological properties. This study highlights the conservation value of ancient woodland and the consequences of landscape connectivity and defragmentation on the genetic setting of a ground beetle. Moreover, it shows that differences in the external shape of male genitalia do not prevent gene flow within the genus *Carabus*. Thus, the establishment of species status should not exclusively be based on this property.

## Introduction

The history of a landscape has a tremendous effect on both the species composition of communities and assemblages on the one hand and the genetic variability of species on the other. This is especially true for woodlands, which have become highly fragmented since the Middle Ages in large areas of north-western Europe including Britain, southern Scandinavia, Belgium, the Netherlands and the lowlands of northern Germany (Desender 2005). Against this background, ancient woodlands, i.e. primary and ancient secondary woods (semi-natural stands and plantations), originating before a threshold date linked to the availability of sufficiently good maps, have a special ecological and historical significance compared to recent woodlands (e.g. Peterken 1993; Rackham 2003). While 1600 AD is used to define ancient woodland in England (Peterken 1977), the first maps available for north-western Germany are from the end of the 17th/beginning of the 18th century (e.g. LeCoq 1805), so that woodlands existing since that time are considered ancient in the sense of Rackham (2003). For north-western Europe, several studies have demonstrated that ancient woodlands host species that do not occur in recent woodlands (for plants: e.g. Peterken 1974; Hermy et al. 1999; Wulf 2004; for animals: e.g. survey in Peterken 1993; Assmann 1999; Desender et al. 1999).

At the genetic level some studies have shown a strong differentiation between remnants of stenotopic woodland species in ancient woodlands that were at least in former times isolated from one another (e.g. Assmann and Günther 2000; Desender et al. 2002; Desender et al. 2005; Drees et al. 2008). Only very few cases are known that have documented that an increase in connectivity after a period of fragmentation in ecological time has had an effect on the distribution, genetic structure and morphology of stenotopic woodland species (Hale et al. 2001; Hale and Lurz 2003; Drees et al. 2008).

In this study we present an example of clinal variability as the result of an increase in the connectivity of a landscape during the last two centuries. We selected a network of woodlands in north-western Germany that stretches between the only two ancient woodland remnants in the region, and studied the genetic variability and differentiation of the woodland specialist *Carabus violaceus* Linné 1758. Due to its flightlessness, this ground beetle has a low power of dispersal. We used two sets of markers for the analysis, morphometric characters and allozymes, in order to determine typical features of the source populations and their geographic distribution within the contact zone. The results are of increased significance, as the study area hosts not only the nominate form but also *Carabus violaceus purpurascens* Fabricius, 1787, which is ranked as a distinct species by some authors (cf. Jeannel 1941; see also Turin et al. 2003). The existence of these two taxa, however, is assumed to go back at least to range changes and isolation during glacial periods (Assmann and Schnauder 1998), like in many European organisms with hybrid zones in Central Europe (e.g. Hewitt 1999). Thus, the amount of genetic differences between both taxa, which is not the main focus of our study, is likely to be the result of a time span of much more than 200 years.

## Material and Methods

### Study area

**Figure 1.**
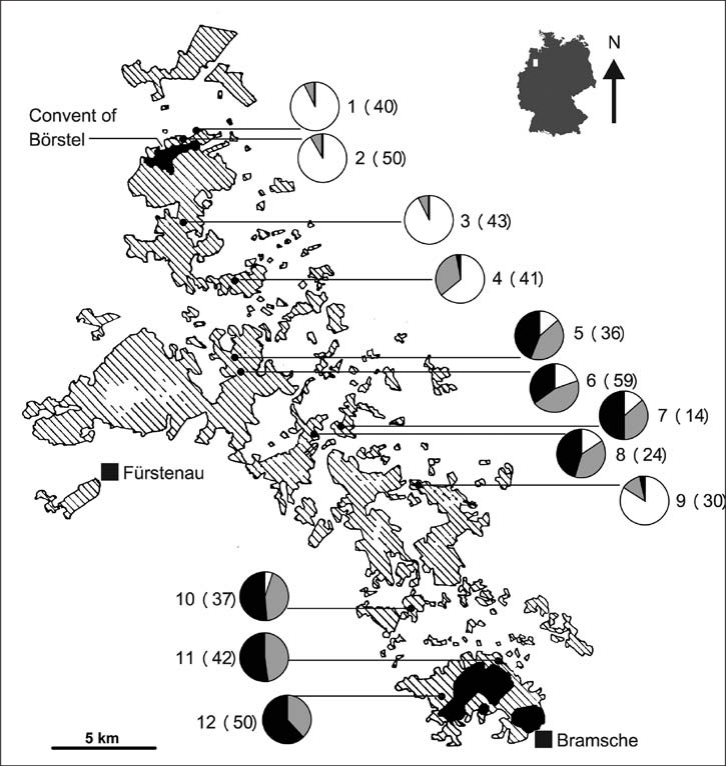
*Carabus violaceus* populations studied and proportion of specimens with different elytron sculptures (pie charts). White sections indicate the frequencies of smooth elytra, black sections indicate the frequencies of more than three striae per elytron, and grey sections indicate the frequencies of intermediate phenotypes, i.e. class „1“. Numbers next to the pie charts indicate population number followed by sample size in brackets. The location of the study area is indicated as a white square on the map of Germany. Woodlands in the study region northwest of the town of Bramsche according to TK 50 3512 Bramsche (Landesvermessungsamt Niedersachsen 1998) are presented as striped patches. Size and position of ancient woodlands (black patches) are taken from the map by LeCoq (1805). In this study, these are called “Börsteler Wald” (in the north) and “Gehn” (in the south). White patches within woodlands indicate openings. Hedges are not shown.

The study area is located northwest of Osnabrück in the morainic hill country between the convent of Börstel and Bramsche (Fig. 1). About 200 years ago the region had only two remnants of woodlands according to the geodesic survey of north-western Germany (LeCoq 1805). These are henceforth called “Börsteler Wald” (north) and “Gehn” (south). The historical situation of the study region is well documented. Excessive heathlands covered the area between the two remnants of (now ancient) woodlands about 200 years ago (Hesmer and Schroeder 1963; Pott and Hüppe 1991). Changes in the socio-economic situation in north-western Germany resulted mainly in the afforestation of heathland and other nutrient poor habitats since *ca*. 1800 (Hesmer and Schroeder 1963; von Oheimb et al. 2008), thus creating a network of recent woodlands that have connected the ancient woodlands Börsteler Wald and Gehn. This is in contrast to many other European countries with temperate climate where the area of woodlands has steadily decreased (Desender 2005). In addition, the forests are connected by a number of hedgerows established over the last two centuries. A comparison of the old and recent situation is exemplified by sections of the original maps in Assmann and Kratochwil (1995: 290 and 291). We studied 466 specimens from 12 populations that were sampled with baited pitfall traps between July and September 1999.

### Study species

*Carabus violaceus* is a flightless woodland species which, in north-western Germany, is more frequently recorded from ancient than from recent woodlands and which is sometimes found in open habitats adjacent to woodlands (Blumenthal 1961; 1965; Dülge 1992; Assmann and Schnauder 1998; Assmann 1999; Falke et al. 2000; Eggers et al. 2010). In the Netherlands, approximately 4 km of open peaty grassland were found to prevent *Carabus violaceus* (subspecies *purpurascens*) from colonizing neighbouring forests, so that the beetle is regarded as highly sensitive to fragmentation of forests (cf. Blumenthal 1981). In our study area, *Carabus violaceus* occurs in both ancient and recent woodlands.

*Carabus violaceus* is an extensive, manifold Euro-Siberian species complex with numerous forms that display slight morphological differences, for example with regards to elytral sculpture, colour and body proportions, and some of which are of doubtful status. Turin et al. (2003) gave an extensive overview regarding current knowledge and discussions on the various forms and their geographic distributions. Two major groups, *Carabus violaceus* s. str. and *Carabus violaceus pupruascens*, are distinguished whose morphological divergence is particularly distinct. The distribution of *Carabus violaceus purpurascens* extends from Germany, Austria and Switzerland westwards to north-western Spain. *Carabus violaceus* s. str. reaches its southwestern distribution in Germany and in parts of the northern Alps and has a Central European to Northern European range up to the British Isles, Scandinavia, the Russian plain close to Moscow, Romania, Bulgaria and Hungary. Combined distribution maps including chorological and taxonomical discussions can be found in Blumenthal et al. (1977) and Assmann and Schnauder (1998). Contact zones exist at least in Germany, Austria and Switzerland.

*Carabus violaceus purpurascens* and the nominate form of *Carabus violaceus* exhibit hybrid populations in north-western Germany (Assmann and Schnauder 1998) and Switzerland (Marggi 1992) that have so far been determined on the basis of the taxonomically relevant differences in elytral sculpture and aedeagus tips. While *Carabus violaceus* s. str. has a broad aedeagus tip and smooth elytra, *Carabus violaceus purpurascens* has a slender aedeagus tip and elytra with distinct ridges (e.g. Henseler 1940; Blumenthal 1976). Isolated populations of *Carabus violaceus* s. str. can be found at least in the Eifel and in the Black Forest (Assmann and Schnauder 1998).

### Allozyme analysis

The abdomina of *Carabus violaceus* (without guts and pygidial glands) were homogenized in 600µl 0.15M Tris-Citrate buffer (pH 7.8, 30% Sucrose, 1% Triton-X-100). After centrifugation, the homogenates were applied to vertical polyacrylamide slab gels and electrophoresis was run at 3°C. Both the mixture of the polyacrylamide slab gels and the staining was performed according to Murphy et al. (1990) with slight modifications (see Appendix 1). From seven enzyme loci screened (AAT, EST-X, GPI, IDH, MPI, PGM, 6-PGD) three were polymorphic and showed interpretable patterns: glucose-6-phosphate isomerase (GPI, Enzyme Commission number 5.3.1.9), mannose phosphate isomerase (MPI, EC 5.3.1.8) and tissue esterase (EST-X, EC 3.1.1.1). Allozymes were numbered in order of increasing anodal migration, and samples were run side by side for comparison (Ayala et al. 1972).

Allele frequencies, observed heterozygosity (*HO*) and mean gene diversity (*HE*) (Nei 1978) were estimated for each locus in each sample using POP100GENE (Piry and Bouget 1999). Tests for Hardy-Weinberg equilibrium were performed with GENEPOP 4.0 (Raymond and Rousset 1995). Data were tested with a probability test (exact HW test) using the Markov chain method. Multiple-testing was corrected for false discovery rate (BL procedure, Benjamini et al. 2001). Tests for genotypic linkage disequilibrium were carried out using FSTAT, V 2.9.3 (Goudet 1995). FSTAT also yielded FST estimates (theta, Weir and Cockerham 1984) and pairwise FST estimates.

Data were investigated for the occurrence of clinal variation by spatial autocorrelation analysis implemented in SGS ver. 1.0 d (Spatial Genetics Software, Degen et al. 2001). This approach tests whether the observed population genetic measure (such as allele frequency) at one sampling site is dependent on the respective measure from samples at neighbouring localities (Barbujani 2000; Manel et al. 2003). A set of genetic distance values increasing from significantly negative to significantly positive scores describes a cline, while values increasing from significantly negative at short distances to insignificant at large distances indicate a pattern of isolation by distance (Chikhi et al. 1998; Barbujani 2000). 6 km-intervals were used as distance classes (distance intervals similar to those chosen for *Poecilus lepidus* by Drees et al. 2010), so that we had five distance classes with between six and 20 data pairs. Confidence intervals were calculated in SGS by running 1000 permutations.

### Morphometric analysis

After material had been taken for allozyme analysis, the animals were placed in Scheerpeltz solution (70% ethanol, 5–10% acetic acid, 15–20% aqua dest.). Male genitalia were prepared and mounted on cards. The remains of the exoskeletons were pinned to dry and deposited in the entomological collection of Thorsten Assmann, Bleckede (to be donated to the Zoological State Collection, Munich).

**Figure 2.**
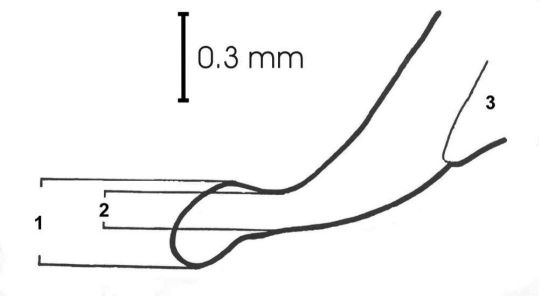
Aedeagus tip of *Carabus violaceus*. **1** Maximum aedeagus width (AedMax), **2** minimum aedeagus width (AedMin), and **3** preputial field.

Two morphological properties that are relevant for taxonomic distinction between both forms of *Carabus violaceus* were analyzed. Since measurements of the aedeagus tips provide suitable characteristics to distinguish *Carabus violaceus* s. str. and *Carabus violaceus purpurascens* (Assmann and Schnauder 1998), we measured the maximum width (AedMax) and minimum width (AedMin) of the aedeagus tip (Fig. 2). Measurements were taken using a stereomicroscope with 25 times 4.0 (ocular times lens) magnification. To keep measurement error at a minimum, parameters for each specimen were measured twice and the termini of the measured lengths were in the same focal plane. Descriptive statistics were performed with STATISTICA Ver. 7.1. We conducted a Kruskal-Wallis-Anova to test for equality of population medians among the groups. Moreover, all populations were tested against each other using the Mann-Whitney-U-test to evaluate differences in aedeagus shape. Multiple testing was corrected for false discovery rate (BL procedure, Benjamini et al. 2001).

As a second morphological character, we used elytron sculpture to classify individuals into the following categories: Class “0” for animals with typically smooth elytra as in the nominate form of *Carabus violaceus*; class “1” for individuals with three striae per elytron; and class “2” for individuals with more than three striae per elytron – as in typical specimens of *Carabus violaceus purpurascens*.

## Results

### Allozyme analysis

A total of 21 alleles were scored at three loci across the 12 populations studied. The number of alleles detected at each locus ranged from five (MPI) to nine (GPI). Allele frequencies, expected and observed heterozygosities and FIS values are shown in Table 1. No significant deviations from Hardy-Weinberg equilibrium were observed for any of the populations or loci after correcting for multiple tests (nominal level of p = 0.05). There seems to be a tendency of populations to display a positive FIS, i.e. a deficit of heterozygotes. However, after correction for multiple testing by Fstat (nominal level of p = 0.05, 720 randomizations), no significant deficit or excess of heterozygotes were found. No significant linkage disequilibrium was found, thus the studied loci can be interpreted as independent markers.

The overall FST value was 0.160 and ranged from 0.127 (GPI) to 0.201 (EST-X). Pairwise population differentiation in FST between the 78 pairs in our study ranged between 0.011 and 0.501, with a significant differentiation for 54 population pairs after standard Bonferroni corrections (Table 2).

**Figure 3.**
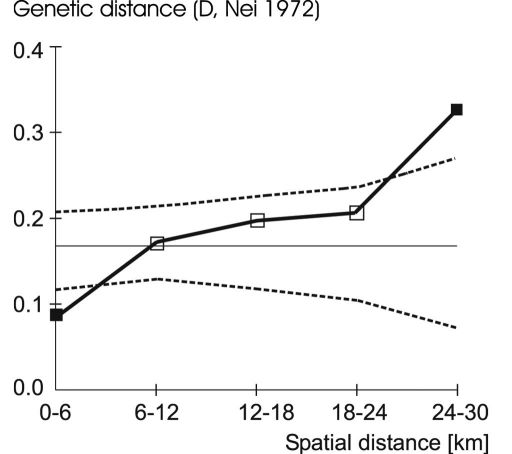
Correlogram showing the result of spatial autocorrelation analysis at three allozyme loci. Genetic distances D (Nei 1972) are indicated for the population pairs of the respective distance classes (squares). Dashed lines show the 95% confidence interval (1000 permutations) under the null hypothesis of spatially random differentiation. Significant deviations from the mean are indicated by filled squares (p < 0.05).

Spatial genetic structure analysis revealed gradients in allele frequencies in the EST-X locus, in the MPI locus, and in the whole sample (Fig. 3, Table 3).

### Morphometric analysis of the elytron sculpture

The vast majority of beetles in the northernmost populations 1 to 4 and population 9 showed the typical smooth elytra of the nominate form, while more than half of the beetles in each of the three southernmost populations (10 – 12) showed the elytron sculpture typical of *Carabus violaceus purpurascens* (Fig. 1). No specimens of *Carabus violaceus purpurascens* were found in the very north and no specimens of *Carabus violaceus violaceus* in the very south. The northernmost individual classified as *Carabus violaceus pupurascens* according to elytron sculpture is one specimen found in population 4, while the southernmost individual classified as *Carabus violaceus violaceus* is one specimen from population 10. All populations contained varying amounts of intermediate individuals of class “1” and, with the exception of population 9, changes in the proportions of different elytron classes are more or less gradual between the ancient woodlands.

### Morphometric analysis of the aedeagus tip

**Figure 4.**
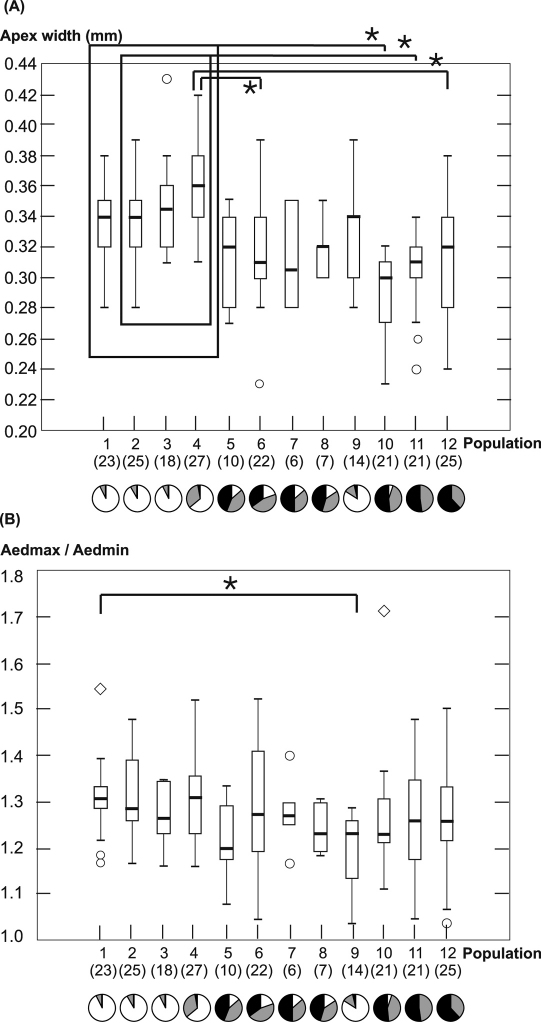
Maximum width of the aedeagus tip **A** and the quotient of maximum and minimum width of the aedeagus tip **B** are plotted for each population. Boxes display 25–75%- quartiles and bars indicate medians. Whiskers show the total range of values without outliers. Outliers are indicated as circles and extreme outliers as diamonds. Numbers of measured individuals per population are shown in brackets. Pie charts show frequencies of elytral sculpture classes “**0**” (white), “**1**” (grey), and “**2**” (black) in each population. Significant differences between populations are indicated by the lines marked with asterisks.

Both the maximum width and the ratio of maximum width to minimum width show significant differences among population medians (AedMax: H(11, N = 220) = 71.157 p < 0.001; AedMax/AedMin: H(11, N = 219) = 22.737 p = 0.019). The pattern of AedMax is very similar to that of the elytron properties. The maximum width of the genital tip is generally highest in populations adjacent to the northern ancient woodland Börsteler Wald, which is characteristic of *Carabus violaceus violaceus*, and smallest in one of the populations close to the southern ancient woodland Gehn, which is characteristic of *Carabus violaceus purpurascens*. Several population comparisons between these two groups display significant differences (Fig. 4). However, the highest and lowest medians of AedMax are not found in the populations directly next to the ancient woodlands, but in populations 4 and 10, respectively, each of which had one specimen with elytron sculpture belonging to the respective other form. Again, we find intermediate values in the forests between the northernmost and southernmost ancient forests, but the overlap of ranges and medians with either populations to the north and to the south is quite high. While the median of AedMax in population 9 is strikingly similar to the medians in the northernmost populations, the median of AedMax/AedMin of population 9 is significantly different from otherwise very similar population 1 (p < 0.05) as AedMin is wider in this population. No other significant difference between populations was found concerning AedMax/AedMin.

## Discussion

### The contact zone

*Carabus violaceus* shows clear gradients both in allozyme markers and morphometric characters in our study area. Clinal variability can be caused by adaptation to geographically changing environmental conditions and by secondary contacts. In the first case, selection is the driving force to create specific patterns of genetic differentiation (e.g. Sezgin et al. 2004; Case et al. 2006). Secondary gradients are the result of evolutionarily neutral processes and describe the situation of a contact zone, which developed after populations had differentiated in disjunct areas. This kind of gradient is a consequence of both range expansion and gene flow (Endler 1977; Hewitt 1988; Kontula and Vainola 2004).

The gradients found in *Carabus violaceus* stretch across an area of recent woodlands, which developed during the last two centuries. Only the outer woodlands are ancient and had the potential to host the woodland restricted beetle during the Middle Ages and early modern times. It is likely that *Carabus violaceus* survived the period of woodland devastation in the ancient woodlands Börsteler Wald and Gehn. Final confirmation that both these areas hosted the source populations for the northern and southern set of characters is still needed. The nearest other potential refuges (ancient woodlands) that are inhabited by *Carabus violaceus* are located several kilometres south and southeast of Bramsche (e.g. Wiehengebirge, Gries et al. 1973; Alt Barenau, Assmann 1999). To the north, there used to be a large peat bog surrounding the Börsteler Wald and there is a gap of at least 20 km in the distribution of *Carabus violaceus* (Gersdorf and Kuntze 1957; Assmann and Schnauder 1998; Assmann 1999). The existence of a different source population at a greater geographic distance cannot totally be ruled out. However, since the area between Börsteler Wald and Gehn was not forested about 200 years ago, the gradients must therefore be secondary. Thus, our study (1) highlights the conservation value of ancient woodland and the consequences of landscape connectivity and defragmentation (*sensu* Hale et al. 2001) on the genetic setting of a ground beetle and (2) yields insights into the evolutionary biology of external male genitalia, gene flow and species delineation.

### A stenotopic woodland ground beetle benefits from habitat defragmentation

Numerous studies have dealt with habitat fragmentation at different levels from populations to whole communities. In general, habitat fragmentation has led to genetic differentiation and extinction processes at the population level and is discussed as one of the driving forces for the loss of species worldwide (Noss et al. 2006; Allendorf and Luikart 2007). Corridors have been discussed as a concept to overcome the problems of habitat fragmentation for some decades (Chetkiewicz et al. 2006), but only a few studies have been able to demonstrate positive outcomes in terms of recovering lost distribution areas or range expansion or recolonizations (Hale et al. 2001; Davies and Pullin 2007).

Our study provides a case of colonization as a result of increased connectivity by means of hedges and afforestation – even though this may not have been the major aim of anthropogenic landscape changes. This is an encouraging example for nature conservation, which generally aims to purposefully reconnect fragmented landscapes. The gradients detected for *Carabus violaceus* cover a distance of approximately 30 kilometres, which is similar to the geographic distances between the postulated source populations of *Carabus auronitens* in the Westphalian Lowlands, NW-Germany, as revealed by both allozymes and microsatellites (Drees et al. 2008). In this species, secondary clines have also developed in a comparable time frame after recolonization of a formerly devastated area. In case of *Carabus auronitens* there is ample evidence that gene flow still exists today (Drees et al. in prep.). Such evidence is strongly dependent on landscape connectivity and history (Desender 2005). In landscapes with more fragmented habitats, where corridors such as hedgerows or small woodlands which can function as stepping stones are lacking, the same species shows strongly differentiated populations without any evidence of recent gene flow (e.g. *Carabus violaceus* in Switzerland: Keller and Largiadèr 2003; Keller et al. 2004; *Carabus auronitens* in Belgium: Desender et al. 2002).

### Excessive gene flow despite differences in aedeagus shape

Elytral sculpture, aedeagus tips and allozymes show that strongly differentiated populations of *Carabus violaceus* survived forest destruction in ancient woodlands within (or close to) the northern and southern edge of the study range. Our results suggest that *Carabus violaceus* s. str. survived in the north, while *Carabus violaceus purpurascens* survived in the south of the study area. These refuges correspond to the overall geographic distribution of the two subspecies (Assmann and Schnauder 1998; Turin et al. 2003).

Elytral sculpture is especially well suited for an unambiguous distinction between both forms, whereas the width of the aedeagus shows significantly differentiated groups, but is a more or less continuously or clinally varying property in the populations. We found many hybrid populations with regards to both properties. Also Assmann and Schnauder (1998) found hybrid populations where numerous individuals showed intermediate characters concerning elytral sculpture and aedeagus shape (two populations were from our study region).

Identifying typical or exclusive alleles for either *Carabus violaceus* form is difficult, as the studied loci generally show clinal variation and as many alleles can be found in populations 1 to 4 as in populations 10 to 12. However, it is likely that the northern refuge population was monomorphic for MDH allele “3”, while the Est-X allele “6” probably originated from a southern population (Table 1). The overall FST value of 0.160 is considerable and shows a fairly high genetic differentiation between the populations in comparison to other organisms studied earlier in this respect, especially with regards to the small geographic scale of the study area (Ward et al. 1992). However, this value lies within the range of “normal” intraspecific variability in habitat specialists of the genus *Carabus* (cf. Matern et al. 2009).

**Table 1.** Diversity of allelic variation. **N** = gene number investigated per sample and per locus; **HO** = observed heterozygosity; **HE** = expected heterozygosity; **FIS** = inbreeding coefficient according to Weir and Cockerham (1984); **NA** = no estimate owing to monomorphic sample.

*Alleles *	*Populations*											
	*1*	*2*	*3*	*4*	*5*	*6*	*7*	*8*	*9*	*10*	*11*	*12*
*EST-X*												
N	70	64	68	46	54	92	22	38	58	48	52	6
1	0	0	0.015	0.022	0.259	0.413	0.091	0.211	0.052	0.042	0.173	0
2	0.714	0.875	0.824	0.783	0.574	0.337	0.182	0.342	0.707	0.146	0.154	0.333
3	0.229	0	0.015	0.043	0.074	0	0	0	0	0	0	0
4	0.057	0.125	0.147	0.087	0.056	0.207	0.682	0.395	0.103	0.354	0.308	0.5
5	0	0	0	0	0	0.011	0	0	0.086	0.25	0	0
6	0	0	0	0.065	0.037	0.033	0.045	0.053	0.052	0.208	0.327	0.167
7	0	0	0	0	0	0	0	0	0	0	0.038	0
HO	0.257	0.25	0.265	0.348	0.37	0.609	0.545	0.684	0.448	0.5	0.462	0.333
HE	0.441	0.222	0.304	0.382	0.604	0.679	0.515	0.698	0.485	0.762	0.758	0.733
FIS	0.420	-0.127	0.132	0.090	0.392	0.105	-0.062	0.021	0.077	0.348	0.396	0.600
*GPI*												
N	76	96	86	80	72	114	28	48	56	70	84	100
1	0	0	0	0	0	0.009	0	0	0	0	0.024	0
2	0	0	0.012	0	0.028	0.018	0	0.083	0	0.043	0.06	0.07
3	0	0	0	0	0	0	0	0	0	0.071	0	0
4	0.263	0.135	0.093	0.225	0.347	0.377	0.536	0.208	0.304	0.257	0.417	0.42
5	0	0	0.058	0.05	0.097	0.096	0	0.063	0.071	0	0.012	0.04
6	0	0	0	0	0	0	0	0	0	0	0	0.01
7	0.697	0.813	0.767	0.65	0.25	0.254	0.179	0.25	0.196	0.414	0.286	0.21
8	0.013	0.01	0.047	0.075	0.278	0.246	0.286	0.396	0.357	0.214	0.202	0.25
9	0.026	0.042	0.023	0	0	0	0	0	0.071	0	0	0
HO	0.421	0.375	0.372	0.375	0.611	0.649	0.5	0.667	0.607	0.714	0.786	0.78
HE	0.449	0.323	0.401	0.525	0.74	0.729	0.622	0.742	0.745	0.72	0.708	0.718
FIS	0.064	-0.163	0.072	0.289	0.176	0.111	0.202	0.104	0.188	0.008	-0.111	-0.088
*MDH*												
N	76	84	84	80	64	108	28	44	56	68	70	66
1	0	0	0	0	0.016	0	0	0	0.161	0.088	0	0.045
2	0	0	0	0	0.047	0.019	0.036	0.182	0.054	0	0.029	0.03
3	1	1	1	0.988	0.906	0.981	0.964	0.818	0.75	0.868	0.714	0.682
4	0	0	0	0	0	0	0	0	0.018	0.044	0.257	0.242
5	0	0	0	0.013	0.031	0	0	0	0.018	0	0	0
HO	0	0	0	0.025	0.188	0.037	0.071	0.364	0.429	0.206	0.371	0.576
HE	0	0	0	0.025	0.178	0.037	0.071	0.304	0.416	0.241	0.429	0.481
FIS	NA	NA	NA	0.000	-0.054	-0.010	0.000	-0.200	-0.032	0.148	0.136	-0.202
*All loci*												
Mean HO	0.226	0.208	0.212	0.249	0.39	0.432	0.372	0.572	0.495	0.473	0.54	0.563
HO SD	0.212	0.191	0.192	0.195	0.212	0.342	0.262	0.18	0.098	0.255	0.218	0.224
Mean HE	0.297	0.182	0.235	0.311	0.507	0.482	0.403	0.582	0.549	0.574	0.632	0.644
HE SD	0.257	0.165	0.209	0.258	0.293	0.386	0.292	0.241	0.174	0.289	0.177	0.142
FIS	0.241	-0.148	0.098	0.200	0.235	0.105	0.078	0.018	0.100	0.178	0.148	0.168

**Table 2.** Significant genetic differentiation (**FST**) between population pairs after standard Bonferroni correction. * indicates a nominal level of p < 0.05; n.s., not significant.

	Pop2	Pop3	Pop4	Pop5	Pop6	Pop7	Pop8	Pop9	Pop10	Pop11	Pop12
Pop1	n.s.	*	n.s.	*	*	*	*	*	*	*	*
Pop2		n.s.	*	*	*	*	*	*	*	*	*
Pop3			n.s.	*	*	*	*	*	*	*	*
Pop4				*	*	*	*	*	*	*	*
Pop5					n.s.	*	n.s.	*	*	*	*
Pop6						n.s.	n.s.	*	*	*	*
Pop7							n.s.	*	*	*	n.s.
Pop8								*	*	*	*
Pop9									*	*	*
Pop10										*	n.s.
Pop11											n.s.

**Table 3.** Spatial autocorrelation analysis of genetic variation at three allozyme loci (multi- and single-locus analysis) in the *Carabus violaceus* populations studied. D values indicate the mean genetic distance observed of samples within each distance class. **–**, D significantly lower; **+**, D significantly greater than the mean genetic distance over all distance classes. ***p < 0.001; **p < 0.01; *p < 0.05; n.s., not significant.

Locus	Distance class [km]				
	0–6	6–12	12–18	18–24	24–30
Pairs of data	16	20	14	11	7
all loci	0.086 (- ***)	0.171 (n.s.)	0.196 (n.s.)	0.206 (n.s.)	0.326 (+ **)
EST-X	0.236 (- *)	0.411 (n.s.)	0.536 (n.s.)	0.657 (n.s.)	0.982 (+ **)
GPI	0.094 (- **)	0.307 (n.s.)	0.346 (n.s.)	0.285 (n.s.)	0.384 (n.s.)
MPI	0.014 (- *)	0.015 (n.s.)	0.030 (n.s.)	0.033 (n.s.)	0.062 (+ **)

The shape of the aedeagus tip is not only used for taxonomic distinction between different carabids, but also for a justification of species rank (Assmann et al. 2008). Some forms of the *Carabus violaceus* complex already have species status, especially because of sympatry without hybridization (e.g. *Carabus violaceus* and *Carabus germari*, Casale and Kryzhanovskij 2003; Turin et al. 2003). For other forms, such as the populations within our study area, there is a discussion on the subspecies or species status. Our study reveals the existence of populations with mixed genomes. Both (1) the lack of a significant deficit of heterozygotes (allozyme markers) within the hybrid zone and at the same time a strong differentiation of the postulated source populations and (2) the concordance of different clines concerning different markers and including numerous individuals with intermediate characters, indicate that the populations are real hybrid populations and not co-occurring species which hybridize occasionally or up to the level of some percentages such as *Carabus auronitens* and *Carabus splendens* in the eastern Pyrenees and *Carabus glabratus* and *Carabus hortensis* in north-western Germany (Assmann 2003). At least occasionally, other *Carabus* species can show higher hybridization rates, e.g. *Carabus lineatus* and *Carabus splendens* in the Val d’Hayra (northern Spain) where, in some years, the proportion of hybrids exceeds 40% (Mossakowski et al. 1986; 1990). However, the difference between these beetles and the hybrid zone of the two *Carabus violaceus* forms is obvious, because the former are co-occurring species which do not show a tendency to “melt down their differentiation” in a hybridization process (own observations two decades after the cited studies).

The existence of a hybrid zone without strong selection pressure (since we found no deviations from Hardy-Weinberg equilibrium after correcting for multiple tests) despite strong differences in the shape of the aedeagus of both forms has consequences for taxonomy within the genus *Carabus*: differences in the male genitalia (especially those of the external shape of the aedeagus tip) do not prevent excessive gene flow and should thus not be used as a character to establish species’ status of forms (cf. Assmann et al. 2008). Moreover, the *a priori* assumption of species-specificity of the genitalia (especially of the external shape of the aedeagus) prevents the detection of species with genitalia differentiated at the subspecies or population levels (Huber 2003). Therefore the establishment of species within the genus *Carabus* should not exclusively be based on differences in the external shape of male genitalia but include other characters such as DNA sequences.

The strong differentiation of the endophallus in some species of the subgenus *Ohomopterus* demonstrates that (1) copulatory pieces can reduce cross-breeding and (2) the fitness costs of interspecific matings are high in the given species (e.g. Sota and Kubota 1998). In these cases the shape of the endophallus and its appendages, not the external shape of the aedeagus, are an excellent character to delineate species within the genus *Carabus*. Moreover, morphological characters to differentiate *Carabus* species should be clear without ambiguity as is the longitudinal striation in *Carabus violaceus purpurascens* in comparison to the smooth elytra of the nominate form.

Numerous questions that are important for a better understanding of the investigated hybrid zone are still unanswered, e.g. if the contribution to gene flow into the hybrid zone is the same for both sexes or if the diffusion rate of markers differs. These and others can only be studied when further molecular, both mitochondrial and nuclear markers are analysed. mtDNA analysis, which is presently being conducted at our institute, may enable us to further estimate evolutionary divergence time between the two investigated subspecies with the help of phylogenetic analysis. The results of the present study reveal that *Carabus violaceus* has the potential to be an important model species in the fields of conservation genetics and evolutionary biology at the interface to systematics.

## Appendix 1

Gel and staining recipes and electrophoresis conditions used in this study. (doi: 10.3897/zookeys.100.1546.app) File format: Adobe Acrobat (pdf).

**Explanation note:** The additional file contains gel, staining recipes and protocols for GPI, MPI and EST-X.

Copyright notice: This dataset is made available under the Open Database License (http://opendatacommons.org/licenses/odbl/1.0/). The Open Database License (ODbL) is a license agreement intended to allow users to freely share, modify, and use this Dataset while maintaining this same freedom for others, provided that the original source and author(s) are credited.
